# Telehealth through the pandemic at a safety net hospital: observations and next steps for cancer care delivery

**DOI:** 10.3389/fpubh.2023.1186350

**Published:** 2023-06-02

**Authors:** Erin Thomas, Alice Kennedy, William Walsh, Michelle Carpentier, Hannah Adeyinka, Shyam Patel, Jonathan Gerber, Jan Cerny, Kriti Mittal

**Affiliations:** ^1^Hospital Medicine, Massachusetts General Hospital, Boston, MA, United States; ^2^Division of Hematology-Oncology, University of Massachusetts Chan Medical School, Worcester, MA, United States; ^3^Division of Hematology-Oncology, University of Massachusetts Memorial Medical Center, Worcester, MA, United States

**Keywords:** telehealth, cancer care, pandemic, COVID-19, safety net hospital

## Abstract

The COVID-19 pandemic revolutionized cancer care delivery leading to rapid adoption of digital technology for telehealth in the United States. In this study, we describe telehealth utilization trends across the three largest waves of the pandemic at a safety net academic center. We also provide a perspective on lessons learnt and our vision for cancer care delivery using digital technology in the near future. The integration of interpreter services within the video platform and its integration within the electronic medical record system is crucial for safety net institutes that service a diverse patient population. Pay-parity for telehealth, especially ongoing support for audio-only visits, will be critical in overcoming health disparities for patients without access to smartphone technology. Use of telehealth in clinical trials, widespread adoption of hospital at home programs, electronic consults for rapid access, and structured telehealth slots in clinic templates will be crucial in making cancer care more equitable and efficient.

## 1. Introduction

The risk of COVID-19 infection continues to pose unique challenges to healthcare delivery for oncology patients. Health safety concerns during the COVID-19 pandemic incentivized the adoption of existing digital technologies in the United States, and worldwide, for remote audio and video consultations. On March 11, 2020, the World Health Organization declared COVID-19 a pandemic (1). According to several U.S. healthcare professional databases, by May 2020, in-office medical visits had declined by as much as 60–70% (2). Claims for oncology office visits fell by 30% in early April 2020 relative to pre-pandemic levels (3). Telehealth gained special importance for cancer patients due to their increased susceptibility to SARS-CoV-2 infection and its complications as oncology patients are often older, have multiple medical comorbidities, and suffer from immunosuppression- factors that are contributory to patients' propensity for severe disease (4, 5). Early studies of cancer patients with SARS-CoV-2, moreover, confirmed that active malignancy and prior exposure to chemotherapy independently increased the risk of death within 30 days of viral symptom onset (6). A large cohort study of 928 cancer patients with SARS-CoV-2 infection identified that those patients with active cancer, advanced age, smoking status, male gender, ECOG ≥ 2, number of comorbidities (2 vs. none), amongst others, were at heightened risk of mortality at 30 days. It is also noteworthy that based on follow up data entry cut off as of May 7, 2020, residence in U.S.-Northeast region was associated with a higher 30-day mortality for patients with past or active diagnosis of cancer compared with those with residence in U.S.-Midwest (odds ratio 0.50; 0.28–0.90) or Canada (0.24, 0.07–0.84) (7).

Leveraging existing technologies and making use of expanded services allowable by federal guidelines therefore deserves undivided attention. Reimbursement has been the primary barrier in the past that limited adoption of telehealth technologies. To this end, the Coronavirus Aid, Relief, and Economic Security (CARES) Act broadened criteria for telehealth services billable as “full visits” under Medicare (8). Some private payers also announced similar expansions (9). Finally, the U.S. Health and Human Services Department decision not to enforce penalties for HIPAA non-compliance also encourages adoption of telehealth in oncology practices (10).

The COVID-19 pandemic has revolutionized cancer care delivery for both inpatient and outpatient oncology care. At the time of writing this article, telehealth has become ingrained in our daily clinic workflows, as well as inpatient consults for SARS-CoV-2 positive patients. Over the next 10 years, the number of cancer survivors living in the United States is expected to increase by 24% to 22.5 million (11). Lessons learned about effective implementation of telehealth for cancer patients during the COVID-19 pandemic, therefore, have important implications for improving care access and cost-savings for this growing population. Below we describe trends in outpatient tele-oncology at our safety-net hospital during the three initial waves of the pandemic, review lessons learned, and envision the next steps in this model of cancer care delivery in resource-constrained settings.

## 2. Methods

Data were collected in aggregate from workbench reports from EPIC electronic medical record system accounting for all outpatient hematology and medical oncology encounters at our primary academic site. Data for weekly statewide incidence of new COVID-19 cases were obtained from the Department of Public Health website (dph.gov) to provide an estimate of COVID-19 case burden in Massachusetts. Inpatient COVID-19 hospitalization metrics at our institution were obtained from our COVID Command Center, to reflect case acuity index of our local patient population. The three pandemic waves for purposes of data collection were defined as: wave 1 (3/2/2020–6/26/2020); wave 2 (10/3/2020–3/27/2021) and wave 3 (12/1/2021–3/4/2022). Telehealth utilization data from our cancer center were then superimposed onto trends of COVID-19 case burden, statewide and within our institution, to contextualize weekly telehealth volumes of the outpatient cancer clinic with the incidence of COVID-19 in our state.

## 3. Results

**Figure 1:**
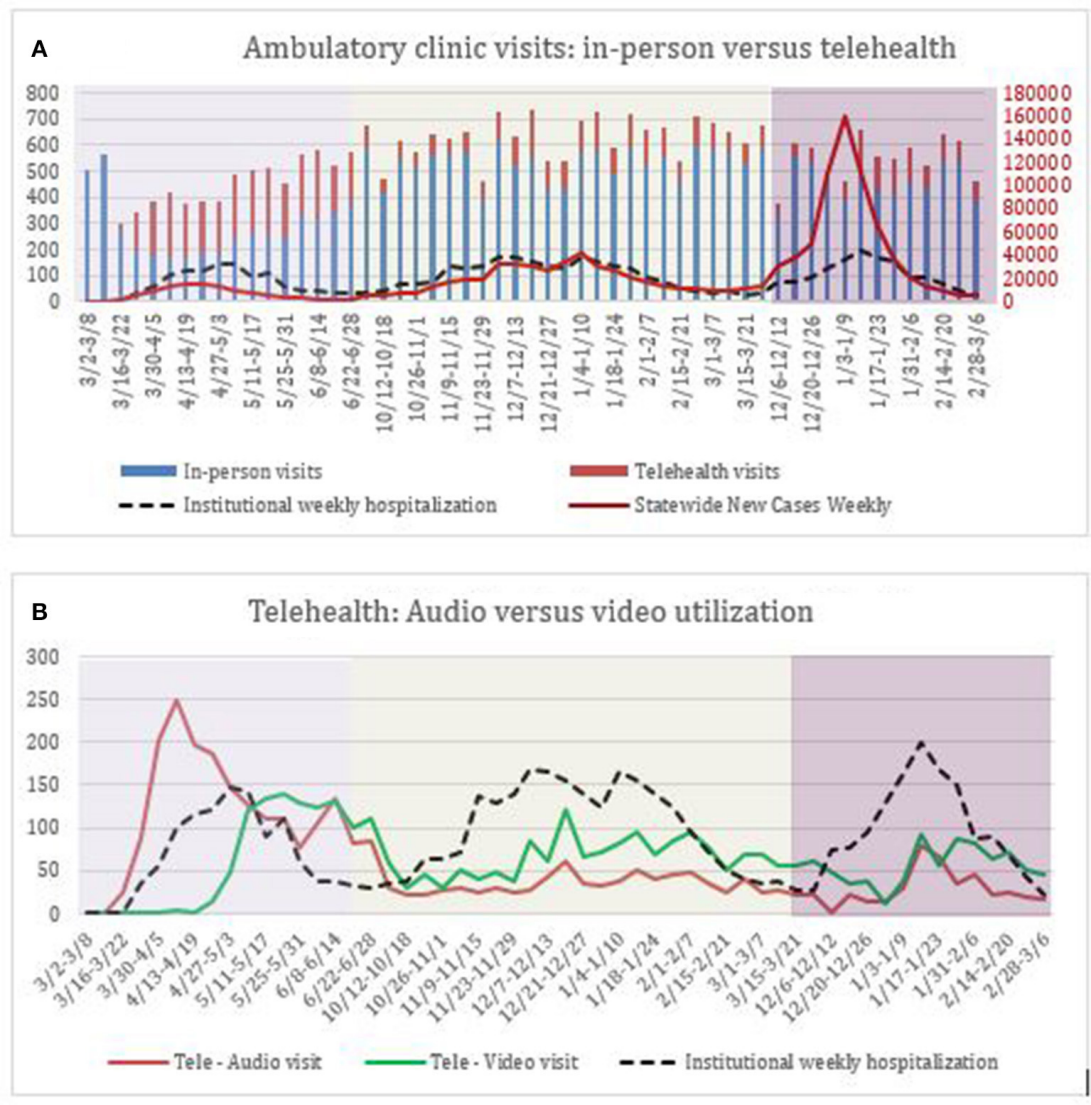
**(A)** Demonstrates weekly visits by type during three waves of the COVID-19 pandemic. *Y*-axis on the left depicts total number of weekly in-person, telehealth oncology visits as well as absolute number of institutional weekly hospitalizations related to COVID-19. *Y*-axis on the right depicts total number of weekly statewide new COVID-19 cases. **(B)** Demonstrates absolute numbers of audio vs. video telehealth visits by week during three waves of the COVID-19 pandemic, with institutional weekly hospitalization rate provided as a reference.

Prior to the March 2020, the Cancer Center at University of Massachusetts Memorial Medical Center (UMMMC) did not offer telehealth visits. During the first week of March 2020, COVID-19 cases began to present locally at our institution. Figure 1A demonstrates the trend in weekly total inpatient admissions at our site during the three largest waves of the pandemic in Massachusetts between 2020 and 2022. The uptrend in our institution's inpatient COVID-19 hospitalizations at the onset of wave 1 was consistent with the increase in statewide new cases of COVID-19. During the first wave, the incidence of weekly institutional COVID-19 admissions peaked at 147 in the week of 4/27/2020–5/3/2020 before down-trending to 29 at the end of wave 1. In parallel, statewide new COVID-19 cases peaked at 15,393 during wave 1 in the week of 4/13/2020–4/19/2020. Telehealth use, as a proportion of total clinic encounters peaked a few weeks prior during the week of 4/6/2020–4/12/2020 when 60% of patient visits were conducted via telehealth. At the tail end of wave 1, telehealth continued to be utilized in lieu of in-person visits by 34% of patients. During waves 2 and 3, the rate of institutional weekly hospitalizations at our site consistently correlated with the statewide incidence of new weekly cases. As clinic volumes recovered during wave 2, the relative proportion of telehealth visits as a percentage of total encounters varied from 10 to 25%, peaking during the week of 12/14/2020–12/20/2020, preceding the peak statewide new case incidence by 3 weeks. At the onset of wave 3 in December 2021, healthcare workers as well as oncology patients had widespread access to COVID-19 vaccines. During this wave, the percentage of telehealth utilization varied from 5 to 26%. The average utilization of telehealth for clinical encounters during waves 1, 2, and 3 was 39, 15, and 15%, respectively.

Figure 1B demonstrates the differential utilization of audio vs. video visits for telehealth encounters. At the onset of the pandemic, audio-visits constituted the vast majority of clinical encounters prior to 4/27/2020 (94–100%). Starting 4/27/2020, a new telehealth platform was adopted system wide that greatly reduced logistical barriers to video consultations and integrated within our electronic medical record system. The conduct of video-visits rose exponentially after implementation of this new platform during wave 1, increasing from 24% of all telehealth visits to 57% at the tail of wave 1. During waves 2 and 3, the use of video visits consistently exceeded the use of audio-only visits. At the tail of wave 3, 73% of telehealth encounters remained video based.

## 4. Discussion

The COVID-19 pandemic accelerated the adoption of novel models of care delivery in oncology, propelling telehealth to the forefront of cancer care since 2020. Some organizations, such as the U.S. Department of Veterans Affairs, expanded the use of pre-existing telehealth initiatives to expand cancer care for rural areas (12) and others developed models for in-home cancer treatments with remote monitoring (13). Given the global nature of the pandemic, it is not surprising that many centers of the world reported changes in practice, incorporating telehealth for up to 75% of cancer care (14–16). The American Society of Clinical Oncology (ASCO) evaluated the adaptations made to cancer care delivery and research in response to this pandemic and published a comprehensive report outlining recommendations on how to make both high quality cancer care and oncological research more accessible, equitable, and efficient going forward (17).

As a safety net academic hospital, our institution serves a county where 10% of the population lives in poverty (18). MassHealth patients account for 26% (internal data) of our clinical volumes. Safety net hospitals, while providing oncologic care to a large number of underserved patients and ethnic minorities, face unique challenges due to resource constraints (19). Our institution's response to local COVID-19 cases included the adoption and implementation of telehealth visits which were previously not offered at our cancer center. Consistent with local demographics (18), 15–20% of oncology patients are non-English speaking and we provide in-person, telephone and video-based interpretation services for all our patients. The integration of interpreter services within our video platform and the electronic medical record system was imperative to successful roll out of telehealth at our site. After the basic workflow was developed by our health informatics team, intensive physician training was conducted, led by a core of volunteer medical students who provided individual and group training as well as elbow support. Continued use of telehealth even after widespread availability of COVID-19 vaccines was noteworthy- with average utilization of 15% in both waves 2 and 3, with peak utilization rate of 25 and 26% during wave 2 and 3, respectively. While patient level data for adoption of telehealth are not available, nonetheless, these trends suggest that this modality remains an important vehicle for cancer care delivery at our safety net site, with the potential to improve health equity by overcoming barriers of transportation access amongst underserved patients. Therefore, it is incumbent upon policy makers to advocate for continued approval of telehealth visits by insurance payers. A recent report of recommendations from the ASCO Global Webinar Series regarding the COVID-19 pandemic emphasized that if telehealth is to be utilized post pandemic, it will require the continued advocacy of specialty societies and bolstered payer and government relations (20).

Our institution implemented both video and audio telehealth modalities at the start of the pandemic. As displayed in Figure 1B, there was a sharp increase in the use of video during the week of 4/27–5/3. This transition coincided with contracting a new telehealth vendor beginning on 4/24/2020, which helped avoid video technology pitfalls which previously caused numerous visits to be converted from video to audio health, demonstrating the importance of user-friendly digital platforms to facilitate telehealth. From this point onwards, most patients opted to conduct their telehealth visits via video. Barring issues with technology, we therefore report a preference for video communication for oncology visits. In its current format, there exists a direct interface between our electronic health record and our preferred telehealth platform. As mentioned in the ASCO Webinar series, interoperability between telemedicine software and the electronic health record has been crucial in efficiently conducting visits with real-time access to medical records and documentation as well as simplifying the scheduling process (20). The rapid adoption of telehealth in our oncology center reflected awareness of cancer patients' susceptibilities to COVID-19. This is a vulnerable population amidst this pandemic due to patients' immunocompromised status, frequent hospitalizations and office visits, and in some cases, poor performance status and transportation barriers.

The expansion in Medicare coverage through multiple stimulus packages in March 2020, including the CARES Act, facilitated the accelerated integration of oncology telehealth services. Prior to this, telehealth services were only covered in non-urban areas or areas with a shortage of healthcare providers. Due to the exceptions made during the pandemic, even new patient telehealth visits have been covered for reimbursement (9). Furthermore, it was decided that penalties for HIPAA violations made in good faith while utilizing telehealth would not be enforced, and remote supervision of oncology services by physicians was also made permissible (10). Together, these factors allowed for the use of both audio-only visits and video visits at the start of the pandemic. Going forward, it remains to be seen what the reimbursement structure will be for telehealth visits, especially for audio-only visits. This will likely be a factor in deciding the longevity of telehealth. Looking ahead, it is predicted that there will be an almost 50% increase in cancer care demand by 2050 due to increasing cancer rates in an aging population and increased survivorship of cancer patients (21). Simultaneously, there will be a shortage of oncologists to meet this need as existing oncologists retire (22), and the pandemic has threatened to exacerbate staffing gaps due to increased clinician burnout (23). Telehealth shows promise in helping to meet this demand by increasing accessibility and efficiency, and in turn, decreasing physician burnout.

It is also pivotal to leverage telehealth in elimination of healthcare disparities in our underserved communities. Advanced age, low literacy, access to video technology, primary language are all potential factors that may conversely impact utilization of telehealth. A recent ASCO report on insights from the pandemic outlines the need for further research into how to optimize technological implementation for telemedicine visits, the need for broadband expansion to provide access for underserved populations, and the importance of adopting new, effective communication styles with patients in the absence of traditional tools like body language in person (17). An early study at UCSF Comprehensive Cancer Center showed that as the proportion of video visits increased as high as 72%, there was not a disparity found based on race/ethnicity, primary language, or payor (24). Finally, we have a responsibility to our patients to make sure that limited direct contact in the office does not fracture the doctor-patient relationship during a vulnerable time in their lives, and the integration of patient surveys could provide helpful insight going forward.

In rural areas, it seems telehealth has improved access to specialty care. Thirty three percent of veterans in the Veterans Health Administration (VHA) system live in rural areas, and the expansion of telehealth has led to decreased wait times to be seen by a specialist, as well as reduction in travel costs and days off from work for patients and caregivers (12). It would be interesting to see if this accessibility has led to decreased disease severity on presentation.

Telehealth may also have long-term adverse effects on clinical trials. An ASCO survey from March 2020 reported that amongst 32 respondents representing both academic and community based programs, about 60% of respondents' programs stopped screening and/or enrollment for certain clinical trials and about 60% halted research-only visits besides those that provided cancer treatment (25). Respondents reported that it was difficult to adhere to clinical trial enrollment guidelines and protocols because of decreased in-person patient visits. With the ongoing use of tele-oncology, it will be important to monitor the impact on experimental therapeutics in oncology in the coming years and perhaps utilize telehealth in the consent, enrollment, and remote monitoring of patients in clinical trials.

How do we envision improvements in tele-oncology to impact cancer care in the next 5 years? Based on the challenges we encountered delivering cancer care in a safety net academic institution during the pandemic, and incorporating lessons learnt in the last 3 years, we propose a potential roadmap in Table 1 to allow for continued adoption of tele-oncology post-pandemic. Anticipated barriers and possible interventions to overcome these barriers are included, Pay-parity for telehealth, especially ongoing support for audio-only visits, will be critical in overcoming health disparities for patients without access to smartphone technology. Industry and co-operative group trials currently do not routinely support trial consents during telehealth visits, and larger conversations around this are ongoing. Our site has successfully rolled out the use of a hospital-at-home program using telehealth, and oncology patients have benefited from this method of care delivery. The use of structured telehealth days and electronic-consults may also help overcome barriers to subspecialty consults for cancer patients.

**Table 1:** Roadmap for tele-oncology post-pandemic.

**Vision/investment**	**Barrier**	**Interventions**	**Returns**
Long term pay parity for telehealth visits	Complex interplay between federal, state and private payor guidelines	Congressional intervention, “An Act of Congress”	• Patient convenience • Reduce patient exposure
Routine use of telehealth in clinical trials	Protocol regulations, IRB regulations, industry buy-in	IRB supported guidelines that allow remote monitoring of vitals, video visits, and local labs for select clinical trials	• Increase trial enrollment in rural areas with lack of access to tertiary care • Reduce disparities as patients with limited means to travel gain benefit from trial enrollment
Hospital at Home programs/at home infusion visits	Limited use at this time, primarily due to lack of awareness and resources outside of limited centers	Education and experience with operationalization for wide-spread establishment of “virtual beds”	• Overcome hospital bed shortage • Minimize patient exposure • Reduce cost of care • Minimize burden on ambulatory infusion chair time
Structured telehealth days	Currently in use at many centers but may have initial logistical/scheduling barriers	Modify clinician templates for dedicated tele-oncology slots	• Streamlined clinics • Availability of dedicated sick visit slots • Social distancing in clinic pods • Overcome room shortage • Helps with staffing shortage (front desk, medical assistants)

## 5. Conclusion

The sustained utilization of telehealth in oncology during the three initial waves of the pandemic at our safety-net academic site offers several unique insights and thought-provoking questions for the future of telehealth. In this study, we show that implementation of a new telehealth platform system-wide, in response to the COVID-19 pandemic, and that was integrated into our electronic heath record, greatly reduced logistical barriers to video consultations and allow improved access to healthcare. This may serve as a model that can be translated nation-wide. While the pandemic has undoubtedly left an indelible impact on the practice of oncology, we hope that ongoing development of telehealth technology will improve the framework of cancer care at an international level.

## Data availability statement

The raw data supporting the conclusions of this article will be made available by the authors, without undue reservation.

## Author contributions

ET, AK, KM, and JC: conception and design. ET, AK, KM, MC, and HA: collection and assembly of data. ET, AK, and KM: data analysis and interpretation. All authors: manuscript writing, final approval of manuscript, and accountable for all aspects of the work.
